# Clade Age and Diversification Rate Variation Explain Disparity in Species Richness among Water Scavenger Beetle (Hydrophilidae) Lineages

**DOI:** 10.1371/journal.pone.0098430

**Published:** 2014-06-02

**Authors:** Devin D. Bloom, Martin Fikáček, Andrew E. Z. Short

**Affiliations:** 1 Department of Ecology & Evolutionary Biology, University of Kansas, Lawrence, Kansas, United States of America; 2 Division of Entomology, Biodiversity Institute, University of Kansas, Lawrence, Kansas, United States of America; 3 Department of Entomology, National Museum, Prague, Czech Republic; 4 Department of Zoology, Faculty of Sciences, Charles University, Prague, Czech Republic; Consiglio Nazionale delle Ricerche (CNR), Italy

## Abstract

Explaining the disparity of species richness across the tree of life is one of the great challenges in evolutionary biology. Some lineages are exceptionally species rich, while others are relatively species poor. One explanation for heterogeneity among clade richness is that older clades are more species rich because they have had more time to accrue diversity than younger clades. Alternatively, disparity in species richness may be due to among-lineage diversification rate variation. Here we investigate diversification in water scavenger beetles (Hydrophilidae), which vary in species richness among major lineages by as much as 20 fold. Using a time-calibrated phylogeny and comparative methods, we test for a relationship between clade age and species richness and for shifts in diversification rate in hydrophilids. We detected a single diversification rate increase in Megasternini, a relatively young and species rich clade whose diversity might be explained by the stunning diversity of ecological niches occupied by this clade. We find that Amphiopini, an old clade, is significantly more species poor than expected, possibly due to its restricted geographic range. The remaining lineages show a correlation between species richness and clade age, suggesting that both clade age and variation in diversification rates explain the disparity in species richness in hydrophilids. We find little evidence that transitions between aquatic, semiaquatic, and terrestrial habitats are linked to shifts in diversification rates.

## Introduction

One of the most remarkable and pervasive patterns on Earth is the uneven distribution of species richness among clades. Indeed, some clades such as beetles are astoundingly species rich, while others such as monotremes are species poor. While there has long been interest in the disparity of species richness across the tree of life [1]–[3], recent advances in comparative methods have made investigating the underlying causes tractable [4]. However, the causes of disparity in species richness among lineages remain controversial [5], [6].

One intuitive explanation for disparity in species richness is that species rich clades are older, and thus have had more time to accumulate diversity than younger clades [7]. This scenario assumes that constant rates of lineage diversification over time result in a predictive positive relationship between clade age and species richness. Disparity in species richness among clades may also result from differences in net diversification rates (speciation minus extinction) among lineages [8]. Diversification rate differences can result from both intrinsic factors (e.g., key innovations) and extrinsic factors (e.g., habitat shifts), or ecological limits (density-dependence) on clade diversity [9], [10]. Widely varying net diversification rates are expected to weaken a clade age-species richness relationship [10], or even decouple age-diversity correlations completely [11].

Studies investigating the disparity of species richness among clades have been met with mixed results [5], [7]. Some studies have failed to detect a relationship between clade age and species richness in groups including plants [12], [13], birds [14], and squamates [8], [15]. Other studies have detected a positive relationship between clade age and species richness in groups such as turtles [16], geckos [17], and diving beetles [18]. In a landmark meta-analysis study, McPeek and Brown [7] analyzed 163 species-level phylogenies and found a positive relationship between clade age and species richness and concluded that species richness in most clades is explained by clade longevity. More recently, using a time-tree of Eukaryotes, Rabosky et al. [5] found no relationship between clade age and species richness across all multi-cellular organisms. Intriguingly, when Rabosky et al. [5] analyzed 12 major subgroups independently (e.g., gymnosperms, mammals), beetles were the only group that showed a positive clade age-species richness relationship. However, when a more densely sampled coleopteran data set was analyzed this positive clade age species relationship did not hold [5]. The generality of this pattern within Coleoptera remains unknown because there have been few studies explicitly investigating the roles of clade age and diversification rates in determining species richness patterns within diverse beetle groups.

**Figure 1:**
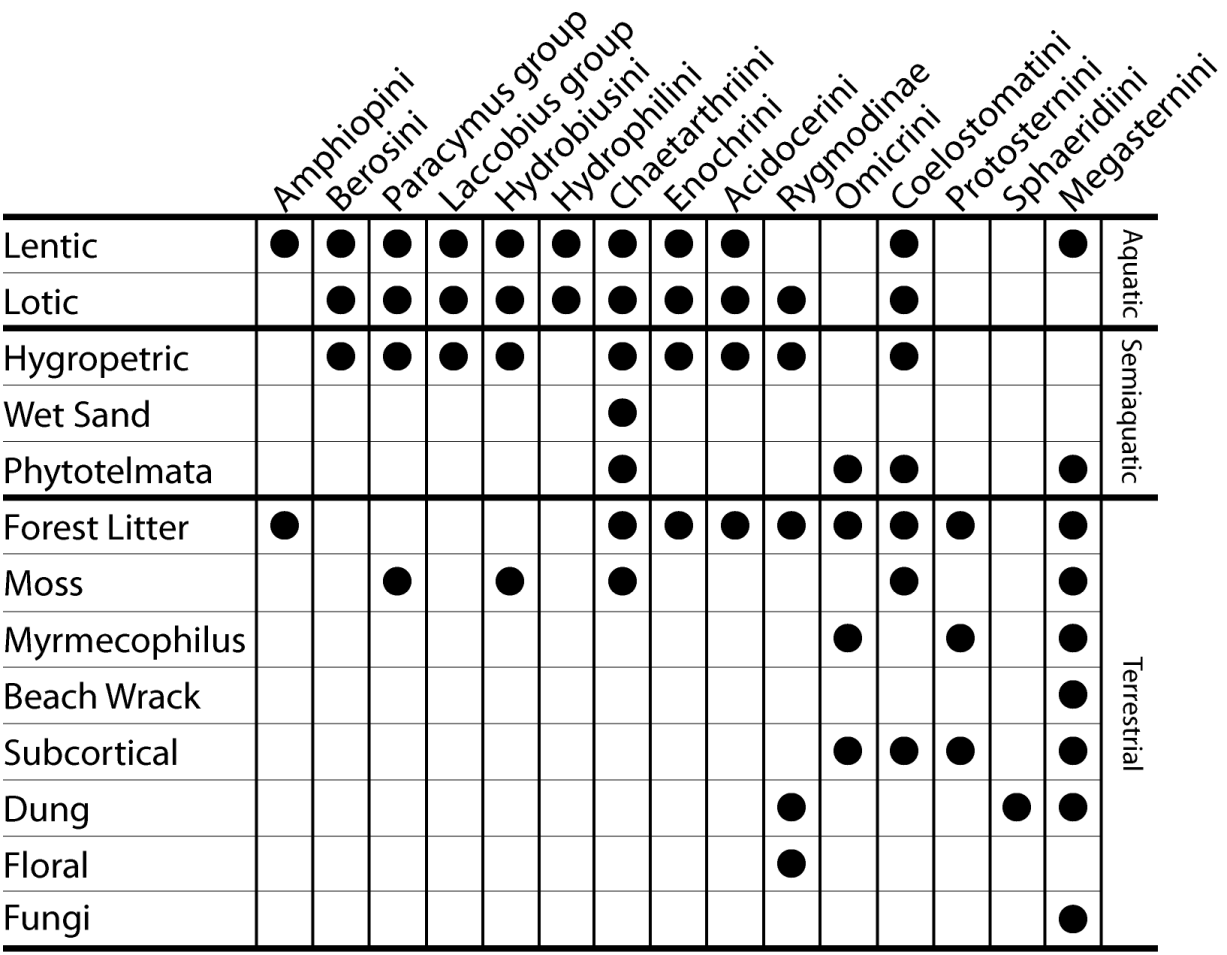
Distribution of habitat types across the major clades of water scavenger beetles.

**Figure 2:**
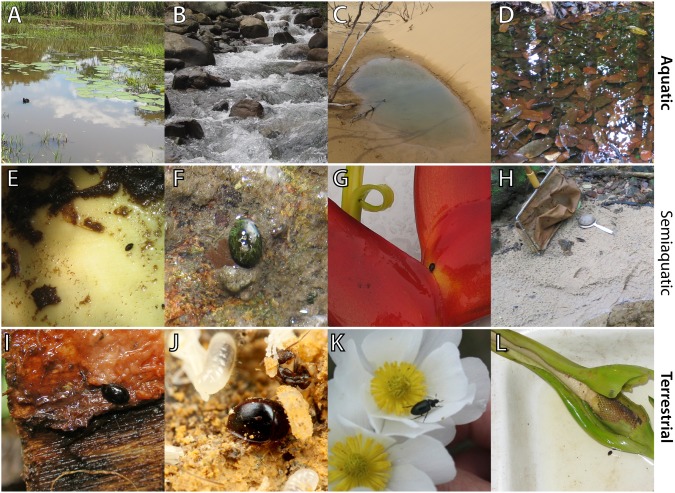
A selection of habitat diversity within the Hydrophilidae. A) Lentic habitat in a vegetated marsh, B) Lotic habitat along a mountain stream, C) Coastal dune pond, D) Detrital forest pool, E) *Anacaena* sp. (Chaetarthriinae) inside a drained bromeliad tank, F) *Oocyclus* sp. (Laccobiini: *Laccobius* group) on a wall seepage, G) *Pelosoma* sp. (Megasternini) emerging from a *Heliconia* inflorescence, H) wet sand habitat along a creek, I) *Dactylosternum* sp. (Coelostomatini) under the bark of a freshly cut tree, J) *Chimaearocyon shimadai* (Megasternini) in the brood chamber of *Pheidole* ants, photo credit: Taku Shimada, K) *Rygmodus* sp. (Rygmodinae) on flowers, photo credit Richard Leschen, L) *Nitidulodes* sp. (Megasternini) on an aroid inflorescence. All photos by A.E.Z. Short unless otherwise indicated.

Here we investigate clade age and diversification rate in water scavenger beetles (Hydrophilidae). Hydrophilids are an excellent group for investigating the processes that determine species richness patterns because they are a diverse group with over 3000 described species and a nearly global distribution [19], and they show a huge disparity in species richness across major lineages. As their name implies, many hydrophilid species occupy aquatic habitats, such as small ponds, stream margins, and wetlands. However, hydrophilids have also diversified across a remarkable array of semiaquatic (or “intermediate”) habitats including waterfalls and seeps, and terrestrial habitats such as dung, flowers, and forest litter (Figs. 1 and 2). Hydrophilids are thought to have repeatedly transitioned between these varied habitats [19]–[21], however the relative number and frequency of habitat transitions is largely unknown.

Habitat has previously been show to influence diversification rates in aquatic beetles, although most studies have focused on microhabitat differences such as lotic and lentic environments [22], [23]. Macrohabitat transitions in beetles have received far less attention, but studies on other taxonomic groups have demonstrated that macrohabitat and ecological dynamics in general can influence diversification rates [24]–[29]. Thus transitions between aquatic and terrestrial habitats may play an important role in determining diversity patterns in hydrophilids. Indeed, the largest terrestrial clade (Sphaeridiinae+Rygmodinae) contains approximately 35% of all hydrophilid species, suggesting that a shift between aquatic and terrestrial habitats may have been a key event [sensu 30,31] that promoted an increase in diversification rates. However, investigations of aquatic-terrestrial habitat shifts across major lineages of insects and the role of these habitat transitions in determining diversity patterns remain understudied.

The absence of a comprehensive time-calibrated phylogeny for hydrophilids has precluded efforts to disentangle the evolutionary processes that explain species richness patterns in this group. Here we use an extensive set of recently revised fossil taxa and relaxed molecular clock analyses to estimate diversification times for water scavenger beetles. We integrate our phylogeny with data on species diversity based on detailed taxonomic expertise of the group and data on habitat preferences of particular taxa largely based on our direct observations in the field. We use comparative methods to investigate the roles of clade age and among-lineage diversification rate variation in determining patterns of species richness in water scavenger beetles, and explore the influence of transitions between aquatic and terrestrial habitats in driving diversification rates.

## Methods

To determine divergence times for hydrophilids we used a six-gene molecular data set of 151 species that included all major lineages of Hydrophilidae [19], and ran a Bayesian relaxed clock analysis with eight fossil calibrations in the program BEAST v1.7.2 [32]. We used the following fossils to calibrate the tree (see online supplementary material for more details on fossil ages and the calibration schemes): *Protochares brevipalpis* (Late Jurassic, Australia) and *Baissalarva hydrobioides* (Early Cretaceous, Russia) [33] *Hydrobius titan* (actually belonging to the genus *Sperchopsis*, Late Eocene, USA; Fikáček et al., unpubl. data); *Limnoxenus olenus* (Latest Oligocene, France) [34]; *Anacaena paleodominica* from Dominican amber (Early Miocene, Dominican Republic) [35], *Helochares* (*Hydrobaticus*) sp. and *Cercyon* sp. from Baltic amber (Eocene, Europe) (Fikáček, unpubl. data and [36]), and *Helophorus paleosibiricus* (Early Cretaceous, Russia) [37]. We used an uncorrelated lognormal tree prior and a birth-death prior for rates of cladogenesis. The dataset was partitioned by gene with partitions unlinked and a GTR model with gamma-distributed rate heterogeneity used for each partition. We ran two analyses for 100 million generations, sampling every 1,000^th^ generation. We verified convergence of parameter estimates and that effective sample sizes were >200 for all parameters using Tracer 1.5 [38]. We combined runs using LogCombiner v1.6.1 [38] and the maximum credibility tree was generated in TreeAnnotator v1.6.1 [38].

To identify shifts in diversification rate we used MEDUSA [39], a comparative method that combines taxonomic and phylogenetic information to fit diversification models using stepwise addition and Akaike Information Criterion (AIC). We accounted for missing species by incorporating our species richness estimates for each major hydrophilid lineage, and pruned the tree to the most terminal clade for which species richness could be confidently estimated (see electronic supplementary material). MEDUSA uses maximum likelihood to fit birth-death, Yule, or a mixed (both birth-death and Yule) diversification models beginning with a single rate model, and using stepwise addition to add models with increasing complexity (i.e. additional rate shifts). Rate shift models are compared using AICc, with more complex models being added until the AICc threshold is no longer met and the single most likely model is selected. Due to the difficulty of estimating extinction rates from molecular data [40], we implemented all three options (birth-death, Yule, and mixed models).

We determined the expected species richness of a clade given a net diversification rate (using background rate from MEDUSA), a relative extinction rate, and clade age [13] using the R package Geiger [41]. We determined the 95% confidence intervals (CI) for models incorporating high (e = 0.90) and low (e = 0.0) extinction rates. The estimated number of species for clades was plotted with the expected diversity estimates to identify clades that have significantly high or low richness given their respective ages.

We used phylogenetic generalized least-squares regression and standard linear regression to test for a relationship between clade age and log-transformed species richness values using the stem clade ages (some clades were represented by a single representative preventing the use of crown ages) from our hydrophilid time tree and current figures for species richness for each major lineage compiled from literature. It is well known that species-rich lineages of insects harbor much greater diversity than is presently described [42]. To account for this undescribed diversity, we combined our taxonomic expertise to estimate expected species richness values for each major lineage and repeated the analyses (see Table S1 in File S1).

To explore the relative number of transitions between aquatic, semiaquatic and terrestrial habitats we conducted ancestral character reconstruction. Our taxon sampling does not allow us to determine the absolute number of transitions between these macrohabitats, but we can assess where across the entire hydrophilid tree transitions have occurred, and couple this with MEDUSA determined diversification rate shifts to explore a possible relationship between habitat type and diversification rates. We coded aquatic, semiaquatic, and terrestrial habitats as discrete, unordered character states. All character reconstructions were conducted on the maximum clade credibility tree from our BEAST analyses. We used maximum likelihood (ML) in Mesquite v2.6 [43] to reconstruct ancestral character states under the Mk model [44].

## Results

**Figure 3:**
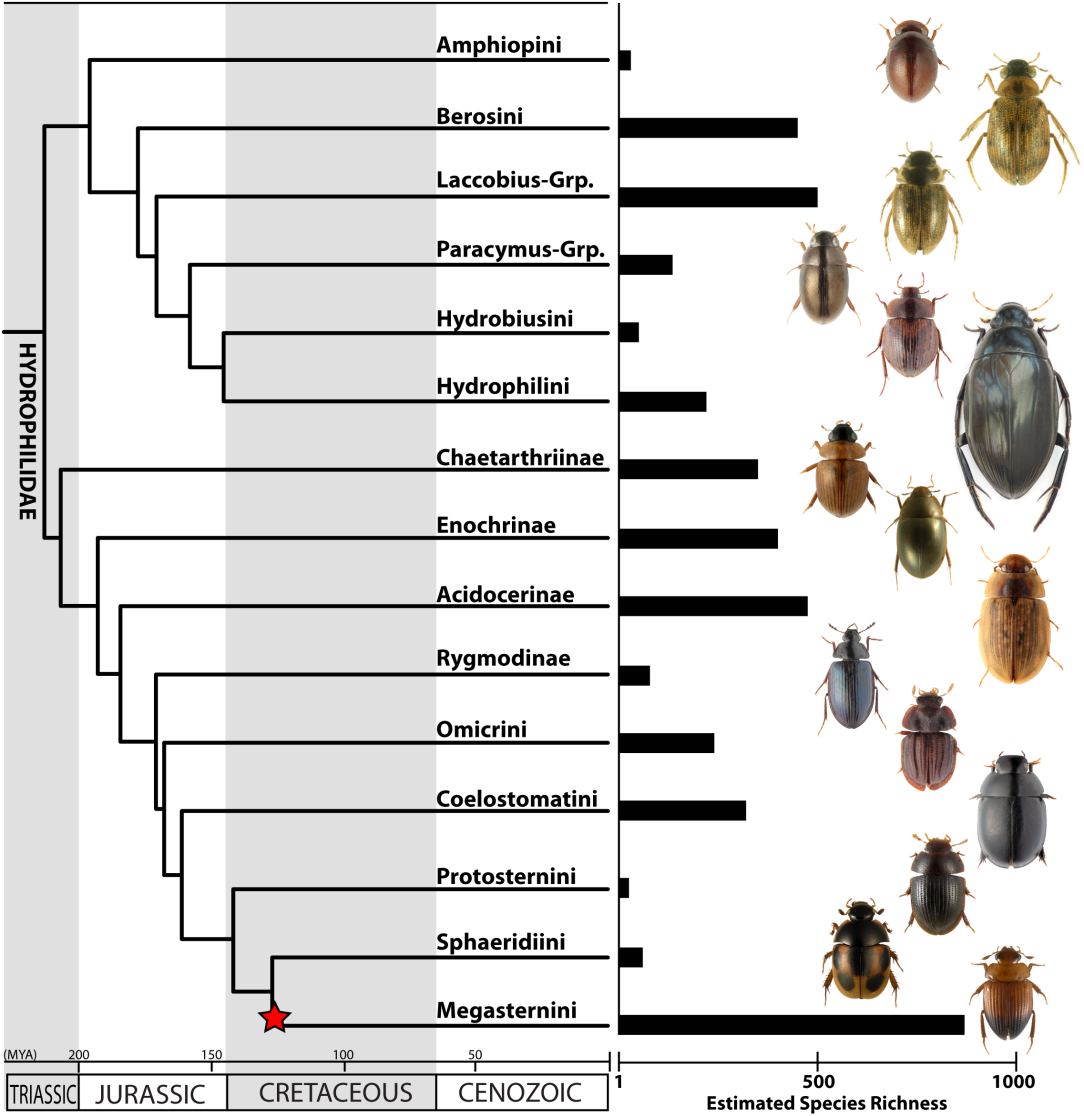
Time-calibrated phylogeny for major lineages of Hydrophilidae, along with estimated species richness values. The star indicates the location of the diversification rate increase determined by our MEDUSA analysis.

We recovered a topology for Hydrophilidae in our relaxed clock analysis that is consistent with Short & Fikáček [19] (Fig. 3, Figures S1, Figures S2, and Figures S3, and Table S2 in File S1). Our divergence time analyses indicate a Late Triassic origin of modern Hydrophilidae (214.1 Ma). Divergence of most major clades (subfamilies and tribes) took place in the Jurassic, with only the two youngest tribes (Megasternini and Sphaeridiini) diverging in the Early Cretaceous (127.5 Ma). Strikingly, one of the youngest clades, Megasternini, is the most speciose major clade of Hydrophilidae.

**Figure 4:**
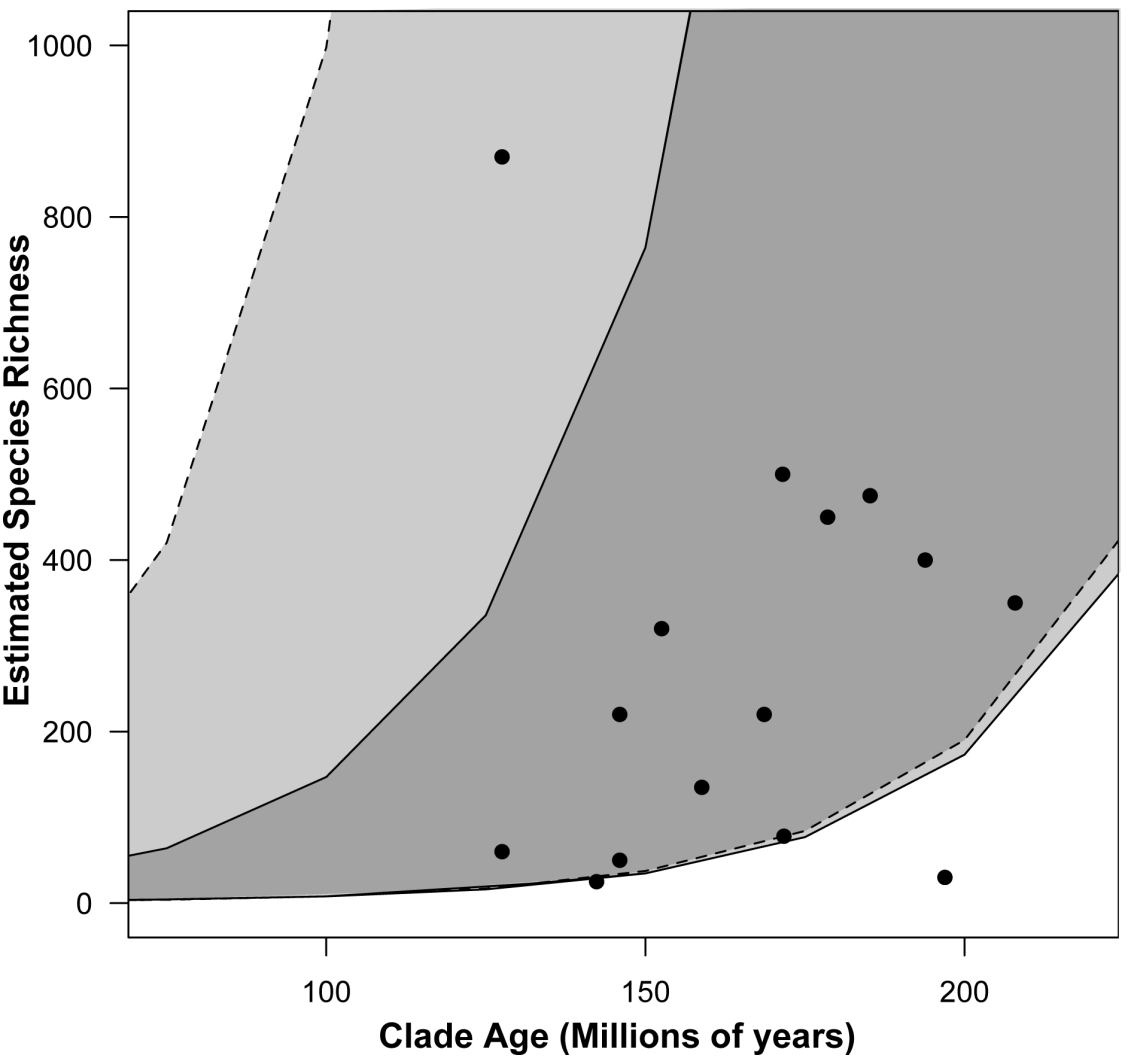
Relationship between clade age and estimated species richness in Hydrophilidae. The shaded regions show 95% confidence interval of the expected diversity under a low (e = 0, solid lines) and high (e = 0.9, dashed lines) extinction.

Our MEDUSA analyses selected a Yule (pure birth) model as the best-fit diversification model. We found a single net diversification rate increase that occurred within the exceptionally diverse Megasternini (r = 0.053) relative to the background rate for hydrophilids (r = 0.032) (Fig. 4). We did not detect any rate decreases across the hydrophilid tree. However, we found that Amphiopini had fewer species than expected (95% CI) under both high and low extinction rate models (Fig. 4). Meanwhile, Megasternini had higher than expected diversity under a model with low extinction rates, but was within the 95% confidence interval of expected species richness under a high extinction rate.

Using phylogenetic least squares regression and standard linear regression we found no relationship between clade age and (estimated) species richness across the full hydrophilid tree (PGLS p = 0.603, linear regression p = 0.520, Fig. 4). However, the removal of Megasternini and Amphiopini (see above) from the dataset results in a significant positive relationship between clade age and species richness (PGLS p = 0.017, linear regression p = 0.009, Fig. 4). We repeated the same analyses using the number of currently described hydrophilids for each major lineage; our major findings were consistent regardless of which species richness values we used (Tables S3, S4, S5 in File S1), therefore we report and discuss the results from the estimated values.

**Figure 5:**
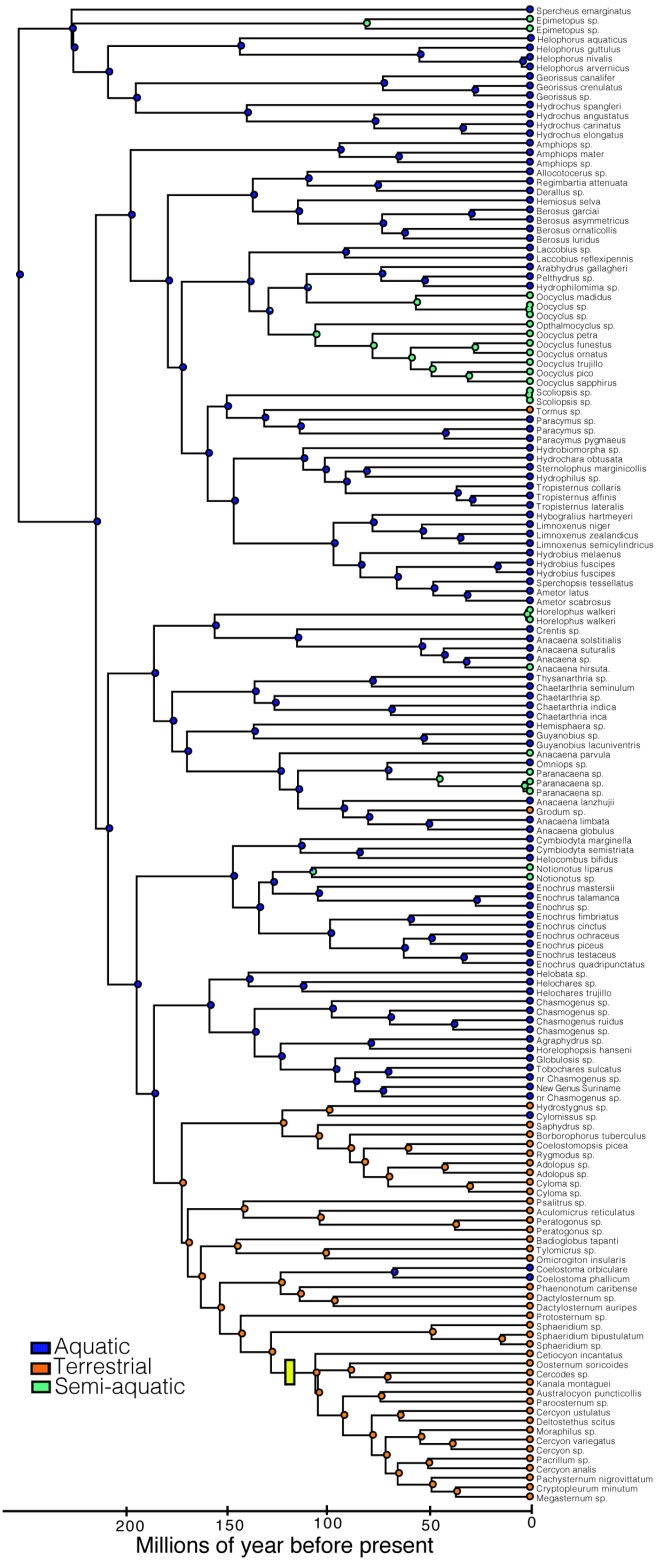
Ancestral character reconstructions of habitat transitions on the time-calibrated phylogeny of Hydrophilidae. The pie charts at nodes indicate maximum likelihood support for ancestral states. A yellow bar indicates the location of the diversification rate shift determined by MEDUSA.

Our ancestral character reconstructions indicate that hydrophilids were ancestrally aquatic (Fig. 5). We infer at least three independent transitions from aquatic to terrestrial habitats, and eight independent transitions from aquatic to intermediate (or semiaquatic) habitats. We infer two secondary returns of terrestrial lineages to aquatic environment. Terrestrial lineages evolved relatively early in the hydrophilid tree, the first instance occurring approximately 171 Ma (Rygmodinae + Sphaeridiinae) and the most recent 80 Ma (*Grodum*-lineage of *Anacaena*). We did not recover any instances where intermediate habitats were a transitional step between aquatic and terrestrial environments.

## Discussion

### Triassic Origins of the Hydrophilidae

Our results show that the origin of Hydrophilidae (214 Ma) considerably predates the estimate found by Hunt et al. [45] for the entire superfamily Hydrophiloidea (175±23.4 Ma), and is more congruent with age estimates for the Hydrophiloidea found by McKenna & Farrell [46] (229–225 Ma). Our molecular age estimates for Early-Middle Jurassic origins of most major hydrophilid clades are consistent with the fossil record because several modern hydrophiloid families were already well established by the Late Jurassic [21], [47] and Hydrophilidae was worldwide in distribution by the Late Jurassic [48]. Thus, our study provides the most robust temporal framework to date for interpreting the diversification of hydrophiloid water beetles.

### Is Disparity in Species Richness Explained by Clade Age or Diversification Rate?

Our study suggests that both clade age and among lineage diversification rate differences explain the disparity of species richness among hydrophilid clades. Our MEDUSA analyses revealed a single increase in diversification rate that occurred in Megasternini. We also found that Amphiopini has fewer species that expected (95% CI) under both high and low extinction rate models given a constant diversification rate (Fig. 4), suggesting this group has unusually low diversity for its relatively old age. However, detecting only a single rate increase suggests that diversification rate variation alone does not explain the disparity in species richness. Our regression analyses show that when hydrophilids are analyzed as a whole, there is a positive, but non-significant relationship between clade age and species richness. When Amphiopini and Megasternini are excluded from the regression analyses (PGLS and standard linear regression), the remaining clades show a significant positive relationship between clade age and species richness (Fig. 4). Thus only two clades, Amphiopini, a relatively old species poor clade, and Megasternini, a relatively young species rich clade, account for the lack of a positive relationship between clade age and species richness. The remaining diversity of hydrophilids shows that species richness can be predicted by clade age; older clades have more species due to greater amounts of time to accumulate lineage diversity.

It is not clear whether net diversification rate or clade age is the predominate factor in determining the pervasive disparity of species richness across the tree of life [5]–[7]. Our study suggests that both factors play a role. However, the majority of diversity within hydrophilids is explained by clade age, supporting the study by McPeek and Brown [7], which showed a strong correlation between clade age and species richness across a wide range of animal taxa. While such studies on beetles are limited to date, there is some evidence that species richness in diving beetles is correlated with clade age [18], suggesting a more general pattern among Coleoptera. When analyzing a time tree representing all Eukaryotes, beetles were one of the very few groups that Rabosky et al. [5] found to exhibit a correlation between clade age and species richness. However, when Rabosky et al. [5] further analyzed beetles using the data set from Hunt et al. [45] they no longer detected a significant relationship between age and richness. It is possible that, much like our results, the latter outcome is driven by a few young exceptionally species rich clades, a few old exceptionally species poor clades, or a combination of the two. If this is the case then beetle diversity may be largely explained by low extinction rates [49] and the ability persisted for a remarkably long time [7], [45].

### Macroecological Shifts and Diversification Rate

Our results support the hypothesis that water scavenger beetles were ancestrally aquatic and have repeatedly shifted between aquatic and terrestrial habitats [20]. However, our results do not support so-called intermediate (semiaquatic) habitats as a transitional step between terrestrial and aquatic states (Fig. 5). Interestingly, transitions from fully aquatic to semiaquatic habitats seem to occur more frequently (eight transitions) than transitions between fully aquatic and terrestrial habitats (five transitions), or between terrestrial habitats and intermediate habitats (zero transitions). This suggests that some transitions such as aquatic to semiaquatic habitats are relatively easy for hydrophilids, but that there are much stronger constraints [50]–[53] on other types of transitions such as re-invading fully aquatic habitats from either semiaquatic or terrestrial habitats. Ribera [54] argued that lentic habitats (e.g., ponds & lakes) select for generalists that are pre-adapted to invade other niches, whereas lotic habitats (e.g., rivers and streams) select for specialists that are unlikely to undergo habitat transitions. Our results suggest that semiaquatic habitats (e.g. seeps, waterfalls, and phytotelmata) also represent highly specialized adaptive peaks, rather than an intermediate stage between aquatic and terrestrial environment. Once a lineage invades this specialized niche it is difficult leave for other regions of the adaptive landscape [55], [56].

We did not detect a diversification rate shift directly associated with any transition between major habitat types (Figs. 3 & 5). Instead, the single rate shift we detected by our MEDUSA analyses followed a major shift from an aquatic-dominant to a terrestrial-dominant lineage by nearly 45 million years (Fig. 5). Furthermore, terrestriality evolved at least two other times (and likely many more in lineages not sampled) in hydrophilid clades that did not experience a diversification rate shift (Fig. 5). This suggests that transitioning into terrestrial habitats did not immediately trigger rapid diversification. However, it is possible that the transition to a terrestrial environment may not immediately spur an increase in diversification, but it sets the stage for a diversification rate shift to occur [57]–[59] by providing the opportunity for rapid diversification in some clades but not others. This might explain the lag time between the origin of terrestriality and the diversification rate shift and why not all terrestrial lineages experienced a rate shift. Whether a trait or evolutionary transition spurs diversification is contingent on interactions with other organisms, traits and the environment [57]. Thus, invading terrestrial habitats may promote diversification, but only in conjunction with other factors. It is also possible that macrohabitat differences do not directly influence net diversification rates to the degree that the use of microhabitats do. If this is the case, it will be necessary to take a more fine scale approach to delineate which terrestrial niches confer elevated rates of diversification.

### Why is Megasternini so Diverse?

With over 540 described species and an estimated 870 species, the Megasternini is remarkably diverse compared to other major hydrophilid clades. This diversity is ultimately the result of an exceptional increase in net diversification rates compared to other hydrophilids (Figs. 3 & 4, supplementary materials). It is difficult to determine if the diversification rate shift is due to an increase in speciation, a decrease in extinction, or both. However, the observed diversity of Megasternini was within the expected diversity for a clade of that age with a high rate of extinction (Fig. 4). One interpretation of this result is that extinction simply has not had enough time to reduce the diversity of this clade (i.e., the pull of the present). However, the selection of a (Yule) pure-birth model suggests extinction rates may not be the driving factor, and that the increase in net diversification is due to increased speciation rates.

Though our results do not implicate a transition from aquatic to terrestrial environments as a cause for explosive diversification within the family, the exceptional species diversity of Megasternini may have an ecological explanation. Megasternini occupies a remarkable diversity of niches within the terrestrial environment and are found in a broader array of habitats than most other hydrophilid lineages (Figs. 2 & 5), such as *Heliconia* inflorescences, leaf litter, ant nests, and mammal dung. For example, the tribe is the only lineage to have significant radiations of myrmecophilous or beach wrack taxa [60], [61]. As habitat has been shown to play an important role in lineage diversification [22], [25], [50], it is possible that the increase in net diversification rate in Megasternini (either a decrease in extinction rate, increase in speciation rate, or both) is linked to the broad spectrum of habitats found in this lineage. Habitats can differ in diversification rates due to various parameters that are associated with that habitat such as the relative presence of barriers, corresponding geographic range size, or some other property linked to habitat [25], [62]–[64]. Alternatively, repeated habitat shifts may have circumvented diversity-dependent regulations of clade growth. The primary mechanism behind diversity-dependence is interspecific competition [11]; the basic concept is that closely related species will compete for limited resources, which in turn reduces speciation and increases extinction and results in a characteristic slowing of lineage accumulation over time [11]. It is possible that repeated transitions between microhabitats allowed Megasternini to escape diversity-dependence by repeatedly presenting ecological opportunity for diversification [9], [65]–[70]. Disentangling which of these mechanisms best explains the remarkable species richness in Megasternini will require dense taxonomic sampling of this clade and to utilize diversification models that explicitly estimate speciation and extinction rates for particular habitats (character states) [71], and to fit diversity dependent diversification models [11], [72].

### Why is Amphiopini Species Poor?

Despite its early Jurassic origin, the Amphiopini is significantly more species-poor than expected under both high and low extinction scenarios given a constant rate (Fig. 4). Our MEDUSA analysis does not detect an exceptional slowdown in diversification rate, and thus begs the question of why the tribe has so few species. One explanation may be that just as the Megasternini exhibits remarkable niche breadth, the ecological diversity within the Amphiopini is atypically narrow for the family. Amphiopini is commonly found in lentic habitats in Africa, Asia, and northern Australia [73]–[75], and represented by a single species found in leaf litter from Madagascar [76]. Lentic habitats are frequently more isolated and more temporary over evolutionary time scales than lotic habitats [54]; these traits have been shown to convey higher rates of dispersal and lower rates of diversification compared to lotic habitats in other aquatic beetle lineages [22]. Thus Amphiopini’s principal specialization in lentic niches may partly explain its relatively low diversity.

Another factor contributing to low species richness in Amphiopini may be its relatively restricted geographic range. Amphiopini is notable in being one of only two major lineages of Hydrophilidae to be absent from the New World. Interestingly, the only tribe that is less diverse (though younger) than the Amphiopini–the Protosternini–also has a relatively restricted geographic range compared to other hydrophilid lineages. It may be that while the diversification rate of Amphiopini has remained similar to the rest of the family, the smaller geographical scale over which it has diversified has limited its absolute species richness.

## Supporting Information

Figure S1 **Hydrophilidae time tree uniform priors.**
(PDF)

Figure S2 **Hydrophilidae time tree exponential priors.**
(PDF)

Figure S3 **Hydrophilidae time tree with posterior support values.**
(PDF)

File S1 **Supplementary materials.**
(DOCX)

File S2 **Genbank vouchers.**
(DOCX)
