# Supplementary material for: Clade Age and Diversification Rate Variation Explain Disparity in Species Richness among Water Scavenger Beetle (Hydrophilidae) Lineages
Source: PLoS One. 2014 Jun 2;9(6):e98430. doi: 10.1371/journal.pone.0098430 (PMC4041770; doi:10.1371/journal.pone.0098430)
Supplement: Figure S2 — Hydrophilidae time tree exponential priors. (PDF) [file pone.0098430.s002.pdf]

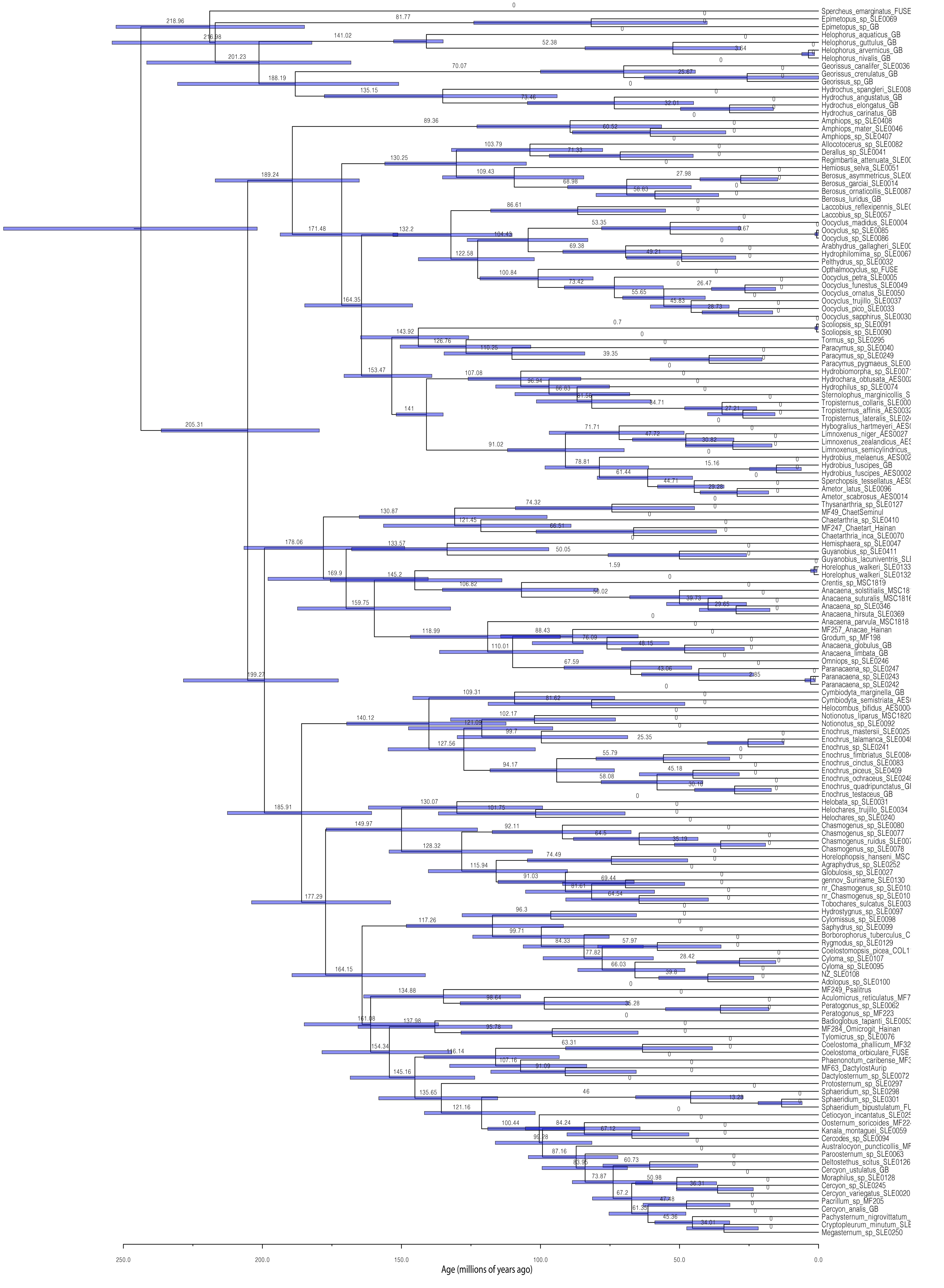

S2. Hydrophilid time calibrated tree using exponential priors showing node ages and 95% HPD error bars.
