# Supplementary material for: Clade Age and Diversification Rate Variation Explain Disparity in Species Richness among Water Scavenger Beetle (Hydrophilidae) Lineages
Source: PLoS One. 2014 Jun 2;9(6):e98430. doi: 10.1371/journal.pone.0098430 (PMC4041770; doi:10.1371/journal.pone.0098430)
Supplement: Figure S3 — Hydrophilidae time tree with posterior support values. (PDF) [file pone.0098430.s003.pdf]

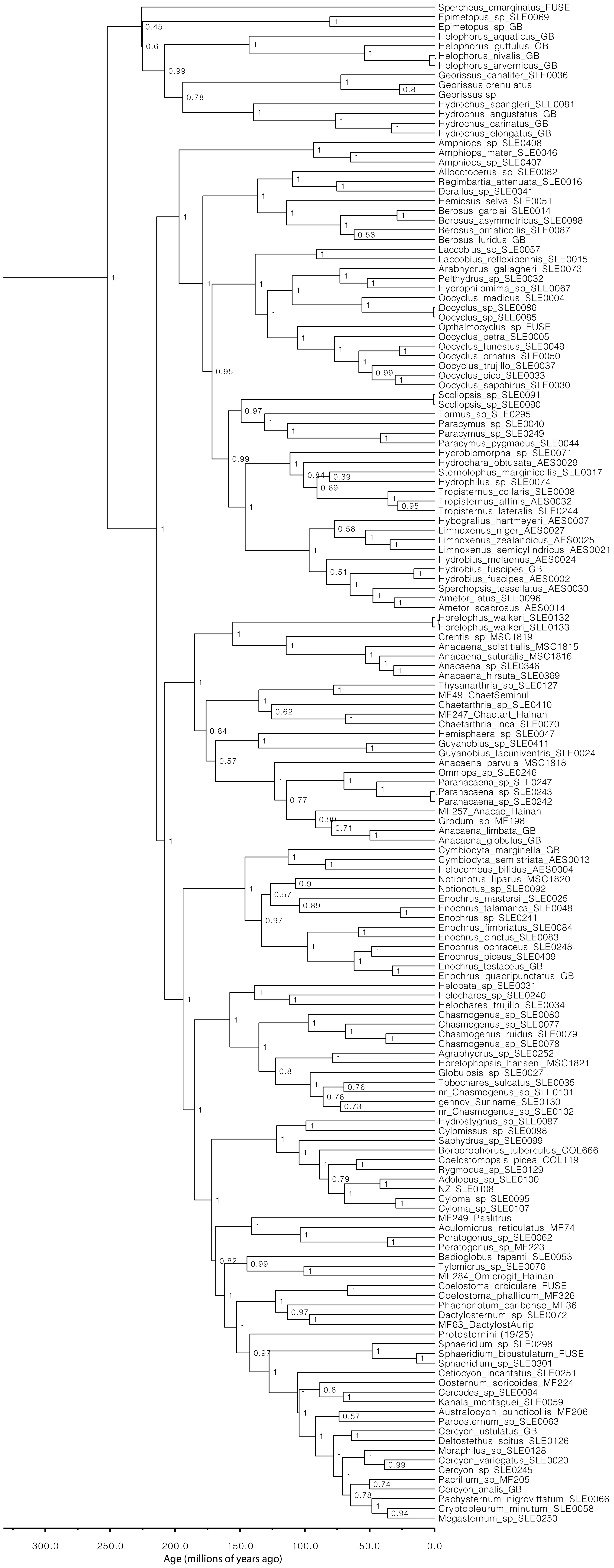

S3. Hydrophilid time calibrated tree using uniform priors showing posterior probability node support values.
