# Supplementary material for: Clade Age and Diversification Rate Variation Explain Disparity in Species Richness among Water Scavenger Beetle (Hydrophilidae) Lineages
Source: PLoS One. 2014 Jun 2;9(6):e98430. doi: 10.1371/journal.pone.0098430 (PMC4041770; doi:10.1371/journal.pone.0098430)
Supplement: File S1 — Supplementary materials. (DOCX) [file pone.0098430.s004.docx]

**Supplementary methods**

**A - Fossil calibrations for diversification time analysis**

The selection of fossil taxa used for the time-calibration points is based on data accumulated during the revision of the fossil record of the superfamily Hydrophiloidea by M. Fikáček and collaborators. In the course of this revision, we have (re)examined the vast majority of the fossils assigned to Hydrophiloidea by previous authors and numerous previously unstudied material.

The following describes the fossil calibration parameters used in the diversification time analyses run in BEAST with uniform priors for all fossil calibrations. We prefer uniform priors because they make the fewest assumptions about the age of a calibrated node except the hard minimum and the hard maximum bounds. However, we also ran the same calibrations using exponential priors with a soft upper bound and recovered younger ages for most clades, suggesting that the hard upper bounds used with uniform priors are not resulting in younger clades ages.

1. *Protochares brevipalpis* is the oldest fossil known that may be reliably assigned to the family Hydrophilidae [1]. It is known from the Talbragar Fossil Fish Bed, which is dated to the Late Jurassic (Oxfordian-Tithonian, i.e. 161-145 mya based on SHRIMP analysis; Kimmeridgian, i.e. 155-150 mya based on fish fauna [[2](#_ENREF_2)]. Based on this fossil, we placed a hard minimum age of 145 mya and a hard maximum of 228 mya on the clade Hydrophilidae (95% credible interval for all upper bounds hereafter). The hard maximum is based on the age estimate for the whole superfamily Hydrophiloidea in the Coleoptera time tree by McKenna & Farrell [[3](#_ENREF_3)] (the Hydrophiloidea clade is erroneously called Hydrophilidae in this publication).
2. *Baissalarva hydrobioides* was demonstrated to belong to the extant tribe Hydrobiusini based on the phylogenetic analysis performed by [4]. The fossil species is known from the Baissa deposits in the Buryat Republic, Russia, which is considered as Early Cretaceous (Berriasian to Hauterivian, ca. 146-135 mya) in age [[5](#_ENREF_5)](Vassilenko, pers. comm.). We used his fossil as a hard minimum bound of 135 mya and a hard maximum of 155 mya (based on the oldest age of the oldest known hydrophilid fossil, *Protochares brevipalpis*, see under 1) for MRCA of Hydrobiusini and Hydrophilini.
3. *Hydrobius titan* described from the Florissant Formation in Colorado, USA, was found to actually represent the extant genus *Sperchopsis* (Fikáček et al., in prep.). The age of the Florissant Formation was revealed as Chadronian in age (37.2–33.9 mya) based on mammal fauna [[6](#_ENREF_6), [7](#_ENREF_7)] dated to 34.1 mya using radiometric dating of volcanic crystals [[8](#_ENREF_8)], and to **33.7-34.7 mya using magnetostratigraphy [**[**6**](#_ENREF_6)**].** Based on this fossil, we set a hard minimum age of 34 mya and a hard maximum of 146 mya (based on the oldest fossil of the hydrobuisini, *Baissalarva*, see under 2) on the MRCA of *Sperchopsis* and *Ametor*.
4. *Limnoxenus olenus* is a member of the extant genus *Limnoxenus* [9] from the Aix-en-Provence Formation in southern France, which is dated to the Latest Oligocene (Late Chattian, ca. 22.5 mya) [9, [10](#_ENREF_10)]. We used *Limnoxenus olenus* to place a hard minimum of 22.5 mya and a hard maximum of 135 mya (based on the oldest fossil of the hydrobuisini, *Baissalarva*, see under 2) for the MRCA of *Limnoxenus*.
5. *Anacaena paleodominica* is known from a well-preserved inclusion in Dominican amber in which many morphological details were available for the identification, including the morphology male genitalia, number of antennomeres and presence of tarsal swimming hairs. This assigned the species to the extant *Anacaena suturalis* species group [11]. Dominican amber is dated back to the Early Miocene (Burdigalian, ca. 19 mya) [[12-15](#_ENREF_12)]. We used *Anacaena paleodominica* to place a hard minimum bound of 19 mya and a hard maximum of 155 mya (based on the oldest age of the oldest known hydrophilid fossil, *Protochares brevipalpis*, see under 1) on the *Anaceana suturalis* group.
6. An undescribed fossil preserved as an inclusion in a piece of Baltic amber may be reliably assigned to the subgenus *Hydrobaticus* of the genus *Helochares* (Fikáček et al. in prep.). Although Baltic amber inclusions are generally found in layers of quite a wide range of age (ca. from Ypresian to Bartonian, 55-37 mya; [[16](#_ENREF_16)]), the main amber horizon (so-called Blue Earth) was dated to Middle Lutetian (ca. 44 mya) using stratigraphy [[17](#_ENREF_17), [18](#_ENREF_18)] and radiometric dating [[19-21](#_ENREF_19)]. Based on this fossil we use a hard minimum of 44 mya and hard maximum of 155 mya (based on the oldest age of the oldest known hydrophilid fossil, *Protochares brevipalpis*, see under 1) on the MRCA of *Helochares* and *Helobata*.
7. A fossil from Baltic amber identified originally as ?*Cercyon* sp. [[22](#_ENREF_22)] was assignment to the Megasternini based on the details of antennal and thoracic morphology (Fikáček et al. in prep.). The dating of Baltic amber is explained above. This Megasternini fossil was used to place a hard minimum of 44 mya and a hard maximum of 155 mya (based on the oldest age of the oldest known hydrophilid fossil, *Protochares brevipalpis*, see under 1) for the MRCA of Megastenini.
8. *Helophorus paleosibiricus* was demonstrated to be a member of Helophorus [23, 24]. This species was found in the Baissa fossil deposits in the Buryat Republic, Russia; see *Baissalarva* for a specification of the age of this fossil site. We used *Helophorus paleosibiricus* to set a hard minimum of 135 mya and a hard maximum of 228 mya on the MRCA of *Helophorus*.

**B - Species richness estimation**

The species richness of clades used in the phylogenetic linear regressions and MEDUSA analyses was determined in two ways: First, we extracted numbers of currently described species from the most current update to the World Catalogue of Hydrophiloidea [[25](#_ENREF_25)]. We accounted for differences between various classification schemes [[26](#_ENREF_26), [27](#_ENREF_27)] by summing richness values at the genus level to match our terminals, rather than using stated richness values for higher-level clades. Because our taxonomic knowledge of major lineages of the family is unevenly distributed (e.g. some clades such as Hydrophilini likely contain very few new species, while other clades such as Anacaenini and Megasternini have experienced significant growth), we used our experience from museum collections and fieldwork to estimate values of the true diversity of each major clade. This estimate is based on the sums of our estimates for each genus of the particular clade.

Table S1. Number of currently described species and the estimated species richness for the principal clades of the Hydrophilidae used for the MEDUSA analyses.

| **Clade** | **Described species**  (Short & Fikáček 2011) | **Estimated** **number of species** |
| --- | --- | --- |
| Amphiopini | 21 | 30 |
| Berosini | 364 | 450 |
| Chaetarthriini | 71 | 100 |
| Hydrobiusini | 45 | 50 |
| Hydrophilini | 198 | 220 |
| Anacaenini | 160 | 250 |
| Laccobiini: *Laccobius* Group | 361 | 500 |
| Laccobiini: *Paracymus* Group | 94 | 135 |
| Enochrinae | 271 | 400 |
| Acidocerinae | 274 | 475 |
| Rygmodinae | 47 | 78 |
| Coelostomatini | 221 | 320 |
| Protosternini | 19 | 25 |
| Omicrini | 104 | 220 |
| Sphaeridiini | 42 | 60 |
| Megasternini | 543 | 870 |

**Supplementary results**

Our analyses provide the first molecular age estimates for the beetle family Hydrophilidae and its subgroups based on the representative taxon sampling of nearly half of all recognized genera covering all taxonomic groups proposed by taxonomists. Our selection of the fossil taxa used for the calibration of the relaxed clock analysis is based on an exhaustive study of the fossil record of the superfamily Hydrophiloidea – all fossils were studied by us and meet the requirements of “best practice for justifying fossil calibrations” sensu Parham et al. [[28](#_ENREF_28)]. Thus our analysis is one of the most rigorous of any beetle timetrees available (see Figure S1).

Table S2. Clade ages with 95% HPD for major lineages of Hydrophilidae from BEAST analyses.

| **Clade** | **Age (Mya)** | **95% HPD (CI)** |
| --- | --- | --- |
| Hydrophilinae | 214.1 | 195– 228 |
| Amphiopini | 196.9 | 176–217 |
| Berosini | 178.6 | 159–197 |
| Laccobius group | 171.5 | 154–190 |
| Paracymus group | 158.8 | 143–175 |
| Hydrobiusini | 146.0 | 137–155 |
| Hydrophilini | 146.0 | 137–155 |
| Chaetarthriinae | 207.9 | 188–224 |
| Enochrinae | 193.8 | 174–211 |
| Acidocerinae | 185.2 | 165–203 |
| Rygmodinae | 171.7 | 153–189 |
| Sphaeridiinae | 171.7 | 153–189 |
| Omicrini | 168.6 | 150–186 |
| Coelostomatini | 152.5 | 134–170 |
| Protosternini | 142.4 | 125–160 |
| Sphaeridiini | 127.5 | 110–144 |
| Megasternini | 127.5 | 110–144 |

Table S3. Results from partial least squares regression analyses on clade age and species richness for both estimated species richness and currently described species richness values from table S1 (above).

|  | t-value | p-value | AIC | logLik |
| --- | --- | --- | --- | --- |
| Full Tree  (estimated richness) | 0.534 | 0.603 | 127.9789 | -60.98943 |
| Megasternini removed  (estimated richness) | 1.485 | 0.163 | 116.4471 | -55.22353 |
| Megasternini & Amphiopini removed (estimated richness) | 2.816 | 0.017 | 102.7126 | -48.3563 |
| Full Tree  (described richness) | 0.382 | 0.709 | 128.0519 | -61.02593 |
| Megasternini removed  (described richness) | 1.263 | 0.231 | 116.9137 | -55.45684 |
| Megasternini & Amphiopini removed  (described richness) | 2.489 | 0.030 | 103.6854 | -48.84272 |

Table S4. Results from standard linear regression analyses on clade age and species richness for both estimated species richness and currently described species richness values from table S1 (above).

|  | t-value | p-value | F-stat | R^2^ (adjusted) | df |
| --- | --- | --- | --- | --- | --- |
| Full Tree  (estimated richness) | 0.662 | 0.520 | 0.438 | -0.042 | 13 |
| Megasternini removed  (estimated richness) | 1.578 | 0.141 | 2.482 | 0.102 | 12 |
| Megasternini & Amphiopini removed (estimated richness) | 3.142 | 0.009 | 9.898 | 0.426 | 11 |
| Full Tree  (described richness) | 0.536 | 0.601 | 0.287 | -0.536 | 13 |
| Megasternini removed  (described richness) | 1.355 | 0.200 | 1.835 | 0.060 | 12 |
| Megasternini & Amphiopini removed  (described richness) | 2.725 | 0.020 | 7.426 | 0.349 | 11 |

Table S5. Results from MEDUSA analysis from both expected and described species richness. The location of the diversification rate shift was identical for both species richness values (shown in figure 1 of main text).

|  | Model | r | r low | R high | LnLik |
| --- | --- | --- | --- | --- | --- |
| Background  (estimated richness) | Yule | 0.033 | 0.030 | 0.036 | -139.1925 |
| Megasternini  (estimated richness) | Yule | 0.053 | 0.041 | 0.075 | -7.7679 |
| Background  (described richness) | Yule | 0.031 | 0.028 | 0.034 | -134.8481 |
| Megasternini  (described richness) | Yule | 0.049 | 0.038 | 0.072 | -7.2962 |

Literature cited

1. Fikáček M., Prokin A., Yan E., Yue Y., Wang B., Ren D., Beattie R. 2014 Modern hydrophilid clades present and widespread in the Late Jurassic and Early Cretaceous (Coleoptera: Hydrophiloidea: Hydrophilidae). *Zool J Linn Soc* **170**(4), 710-734. (doi:10.1111/zoj.12114).

2. Beattie R.G., Avery S. 2012 Palaeoecology and palaeoenvironment of the Jurassic Talbragar Fossil Fish Bed, Gulgong, New South Wales, Australia. *Alcheringa* **36**, 451-465.

3. McKenna D., Farrell B. 2009 Beetles (Coleoptera). In *The Timetree of Life* (eds. Hedges S.B., Kumar S.), pp. 278–289, Oxford University Press.

4. Fikáček M., Prokin A., Yan E., Yue Y., Wang B., Ren D., Beattie R. Submitted Modern hydrophilid beetles present and widespread in Late Jurassic and Early Cretaceous (Coleoptera: Hydrophiloidea: Hydrophilidae). *Zool J Linn Soc*.

5. Zherikhin V.V., Mostovski M.B., Vršanský P., Blagoderov V.A., Lukashevitsh E. 1998 The unique Lower Cretaceous locality Baissa and other contemporaneous fossil sites in North and West Transbaikalia. . In *Proceedings of the first international paleoentomological conference* (pp. 185–191. Moscow, AMBA Projects International.

6. Prothero D.R., Sanchez F. 2004 Magnetic stratigraphy of the upper Eocene Florissant Formation, Teller County, Colorado. In *Paleogene Mammals* (eds. Lucas S.G., Zeigler K.E., Kondrashov P.E.), pp. 129-135, New Mexico Museum of Natural History and Science Bulletin.

7. Lloyd K.J., Worley-Georg M.P., Eberle J.J. 2008 The Chadronian mammalian fauna of the Florissant Formation, Florissant Fossil Beds National Monument, Colorado. In: Meyer H. W. & Smith D. M. (eds.) Paleontology of the Upper Eocene Florissant Formation, Colorado. . *Geol Soc Am Spec Pap* **435**, 117-125.

8. Evanoff E., McIntosh W.C., Murphey P.C. 2001 Stratigraphic Summary and 40Ar/39Ar Geocrhonology of the Florissant Formation, Colorado. In: (eds.) Fossil Flora and Stratigraphy of the Florissant Formation, Colorado. . In *Proceedings of the Denver Museum of Nature and Science* (ed. Evanoff E. G.-W.K.M.J.K.R.), pp. 1-16.

9. Fikáček M., Prokop J., Nel A. 2010 Fossil water scavenger beetles of the subtribe Hydrobiusina (Coleoptera: Hydrophilidae) from the Upper Oligocene locality of Aix-en-Provence. . *Acta Entomol Mus Natl Pragae* **50**, 445-458.

10. Nury D., Thomassin B.A. 1994 Paléoenvironnements tropicaux marins et lagunaires d’un littoral abrité (fonds meubles à bancs coralliens, lagune évaporitique) à l’Oligocène terminal (région d’Aix-Marseille, France). In *Interim Colloquium, Regional Committee on Mediterranean Neogene Stratigraphy (RCMNS)* (pp. 45-46. Marseille, Regional Committee on Mediterranean Neogene Stratigraphy.

11. Fikáček M., Engel M.S. 2011 An aquatic water scavenger beetle in Early Miocene amber from the Dominican Republic (Coleoptera: Hydrophilidae). *Annales Zoologici* **61**(4), 621-628.

12. Iturralde-Vinent M.A. 2001 Geology of the amber-bearing deposits of the Greater Antilles. *Caribb J Sci* **141–167**(37).

13. Iturralde-Vinent M.A., MacPhee R.D.E. 1996 Age and paleogeographical origin of Dominican amber. *Science* **273**, 1850–1852.

14. Iturralde-Vinent M.A., MacPhee R.D.E. 1999 Paleogeography of the Caribbean region: Implications for Cenozoic biogeography. *Bulletin of the American Museum of Natural History* **238**, 1–95.

15. Penney D. 2010 Dominican amber. In *Biodiversity of fossils in amber from the major world deposits* (ed. Penney D.), pp. 22-41. Manchester, Siri Scientific Press.

16. Ritzkowski S. 1999 Das geologische Alter der bernsteinfuehrenden Sedimente in Sambia (Bezirk Kaliningrad), bei Bitterfeld (Sachsen-Anhalt) und bei Helmstedt (SE-Niedersachsen). In *Investigations into Amber: Proceedings of the International Symposium* (pp. 33-40.

17. Kosmowska-Ceranowicz B., Müller C. 1985 Lithology and calcareous nannoplankton in amberbearing Tertiary sediments from boreholes Chlapowo (northern Poland). *Bulletin of the Polish Academy of Sciences* **33**, 119–128.

18. Kosmowska-Ceranowicz B. 1987 Charakterystika mineralogiczno-petrograficzna bursztynonosnych osadów Eocenu w okolicach Chlapowo oraz osadów Paleogenu Polnocnej Polski. *Biuletyn Instytutu Geologicznego* **356**, 29-50.

19. Ritzkowski S. 1997 K-Ar-Alterbestimmungen der bernsteinfuehrenden Sedimente des Samlandes (Palaeogen), Bezirk Kaliningrad. *Metalla* **66**, 19-23.

20. Grimaldi D., Engel M.S. 2005 *Evolution of the Insects*. Cambridge, Cambridge University Press; xv+755 p.

21. Weitschat W., Wichard W. 2010 Biodiversity of fossils in amber from the major world deposits. In *Baltic Amber* (ed. Penny D.), p. 303. Manchester, Siri Scientific Press.

22. Kubisz D. 2000 Fossil beetles (Coleoptera) from Baltic amber in the collection of the Museum of Natural History of ISEA in Kraków. *Polish Journal of Entomology* **69**, 225-230.

23. Fikáček M., Prokin A.A., Angus R.B., Ponomarenko A.G., Yue Y., Ren D., Prokop J. 2012 Revision of Mesozoic fossils of the helophorid lineage of the superfamily Hydrophiloidea (Coleoptera: Polyphaga). *Acta Entomol Mus Natl Pragae* **52**, 89–127.

24. Fikáček M., Prokin A.A., Angus R.B., Ponomarenko A.G., Yue Y., Ren D., Prokop J. 2012 Phylogeny and the fossil record of the Helophoridae reveal Jurassic origin of modern hydrophiloid lineages (Coleoptera: Polyphaga). *Syst Entomol* **37**, 420-447.

25. Short A.E.Z., Fikáček M. 2011 World catalogue of the Hydrophiloidea (Coleoptera): additions and corrections II (2006-2010). *Acta Entomol Mus Natl Pragae* **51**(1), 83-122.

26. Hansen M. 1999 *Hydrophiloidea (s.str.) (Coleoptera).* . Stenstrup, Apollo Books; 416 p.

27. Short A.E.Z., Fikáček M. 2013 Molecular Phylogeny, Evolution, and Classification of the Hydrophilidae (Coleoptera). *Syst Entomol* **38**, 723-752.

28. Parham J.F., Donoghue P.C.J., Bell C.J., Calway T.D., Head J.J., Holroyd P.A., Inoue J.G., Irmis R.B., Joyce W.G., Ksepka D.T., et al. 2012 Best Practices for Justifying Fossil Calibrations. *Syst Biol* **61**(2), 346-359. (doi:10.1093/sysbio/syr107).
