# Supplementary material for: Clade Age and Diversification Rate Variation Explain Disparity in Species Richness among Water Scavenger Beetle (Hydrophilidae) Lineages
Source: PLoS One. 2014 Jun 2;9(6):e98430. doi: 10.1371/journal.pone.0098430 (PMC4041770; doi:10.1371/journal.pone.0098430)
Supplement: File S2 — Genbank vouchers. (DOCX) [file pone.0098430.s005.docx]

**Table.** List of Genbank accession numbers that were used in the phylogenetic analyses.

| **Species** | **CO1** | **CO2** | **16S** | **18S** | **28S** | **ArgK** |
| --- | --- | --- | --- | --- | --- | --- |
| Aculomicrus reticulatus | - | KC992396 | - | KC934982 | KC992514 | - |
| Adolopus sp. | KC935209 | KC992510 | - | KC934983 | KC992515 | KC935108 |
| Adolopus sp. | KC935210 | KC992511 | - | KC934984 | KC992516 | KC935109 |
| Agraphydrus sp. | KC935211 | - | KC992640 | KC934985 | - | KC935110 |
| Allocotocerus yalumbaboothbyi | - | KC992402 | KC992641 | KC934986 | KC992517 | KC935111 |
| Ametor latus | KC935212 | KC992498 | - | KC934987 | - | KC935112 |
| Ametor scabrosus | KC935213 | KC992499 | - | KC934988 | KC992518 | - |
| Amphiops mater | KC935214 | KC992462 | - | KC934989 | KC992519 | KC935113 |
| Amphiops sp. | KC935215 | KC992461 | KC992642 | KC934990 | KC992520 | - |
| Amphiops sp. | KC935216 | KC992463 | KC992643 | KC934991 | KC992521 | - |
| Anacaena sp. | KC935224 | KC992451 | - | KC934999 | KC992529 | - |
| Anacaena lineata | KC935296 | KC992500 | KC992645 | KC934996 | KC992526 | KC935114 |
| Anacaena lindi | KC935219 | KC992501 | - | KC934994 | KC992524 | KC935115 |
| Anacaena lindi | KC935220 | KC992502 | - | KC934995 | KC992525 | KC935116 |
| Anacaena lucida | KC935221 | - | KC992646 | KC934992 | - | KC935117 |
| Anacaena globulus | AM287086 | AM287108 | AM287064 | AM287125 | - | - |
| Anacaena hirsuta | KC935217 | KC992482 | KC992644 | KC934993 | KC992522 | - |
| Anacaena lanzhujii | KC935218 | KC992455 | - | KC935002 | KC992523 | - |
| Anacaena limbata | DQ155734 | - | - | AY745586 | - | - |
| Anacaena parvula | KC935222 | - | - | KC934997 | KC992527 | KC935118 |
| Anacaena solstitialis | KC935223 | - | KC992647 | KC934998 | KC992528 | KC935119 |
| Anacaena sp. | KC935225 | KC992483 | KC992648 | KC935000 | KC992530 | - |
| Anacaena suturalis | KC935226 | - | KC992649 | KC935001 | KC992531 | KC935120 |
| Arabhydrus gallagheri | KC935227 | KC992449 | KC992650 | KC935003 | KC992532 | - |
| Australocyon puncticollis gr. | KC935228 | - | - | KC935004 | KC992533 | - |
| Badioglobus tapanti | KC935229 | KC992467 | - | KC935005 | KC992534 | KC935121 |
| Berosus asymmetricus | KC935230 | KC992440 | KC992651 | KC935006 | KC992535 | KC935122 |
| Berosus garciai | KC935231 | KC992441 | KC992652 | KC935007 | KC992536 | - |
| Berosus luridus | AM287087 | AM287109 | AM287065 | AJ810721 | AJ810756 | - |
| Berosus ornaticollis | - | KC992424 | KC992653 | KC935008 | KC992537 | KC935123 |
| Borborophorus tuberculus | KC935232 | KC992409 | - | KC935009 | - | - |
| Cercyodes laevigatus | KC935233 | KC992504 | - | KC935010 | KC992538 | KC935124 |
| Cercyon analis | DQ156007 | - | - | EF213786 | - | - |
| Cercyon ustulatus | DQ155947 | AM287115 | AM287071 | AM287129 | AM287137 | - |
| Cercyon variegatus | KC935234 | KC992484 | KC992654 | KC935011 | KC992539 | - |
| Cercyon versicolor | KC935235 | - | - | KC935012 | - | - |
| Cetiocyon incantatus | KC935236 | KC992454 | - | KC935013 | KC992540 | KC935125 |
| Chaetarthria inca | - | KC992479 | KC992655 | KC935014 | KC992541 | KC935126 |
| Chaetarthria indica gr. | KC935237 | KC992478 | - | - | KC992543 | - |
| Chaetarthria seminulum | KC935238 | KC992503 | - | KC935015 | KC992542 | - |
| Chaetarthria sp. | KC935239 | KC992429 | KC992656 | KC935016 | KC992544 | - |
| Chasmogenus ruidus | KC935240 | KC992469 | KC992657 | KC935017 | KC992545 | KC935127 |
| Chasmogenus sp. | KC935241 | KC992468 | KC992658 | - | KC992546 | KC935128 |
| Chasmogenus sp. | KC935242 | KC992470 | KC992659 | KC935018 | KC992547 | KC935129 |
| Chasmogenus sp. | KC935243 | KC992466 | - | KC935019 | KC992548 | KC935130 |
| Coelostoma orbiculare | AM287094 | AM287116 | AM287072 | EF213785 | KC992549 | KC935131 |
| Coelostoma phallicum | KC935244 | KC992398 | - | KC935021 | KC992550 | - |
| Coelostomopsis picea | KC935245 | KC992507 | - | KC935022 | KC992551 | - |
| Crentis (s.str.) sp. | KC935246 | KC992413 | KC992660 | KC935023 | KC992552 | - |
| Cryptopleurum minutum | KC935248 | KC992491 | - | KC935024 | KC992553 | KC935132 |
| Cryptopleurum subtile | KC935247 | - | - | KC935025 | - | - |
| Cyloma sp. | KC935249 | - | KC992661 | KC935026 | KC992554 | KC935133 |
| Cyloma sp. | KC935250 | KC992506 | KC992662 | KC935027 | KC992555 | KC935134 |
| Cylomissus glabratus | KC935251 | KC992490 | KC992663 | KC935028 | KC992556 | KC935135 |
| Cymbiodyta marginella | AM287088 | AM287110 | - | AM287126 | AM287134 | - |
| Cymbiodyta semistriata | KC935252 | - | - | KC935029 | KC992557 | KC935136 |
| Dactylosternum auripes | KC935253 | KC992404 | - | KC935030 | KC992558 | - |
| Dactylosternum sp. | KC935254 | KC992400 | KC992664 | KC935031 | KC992559 | KC935137 |
| Deltostethus scitus | KC935255 | KC992480 | KC992665 | KC935032 | KC992560 | KC935138 |
| Derallus sp. | KC935256 | KC992411 | KC992666 | KC935033 | KC992561 | KC935139 |
| Enochrus cinctus | KC935257 | KC992464 | KC992667 | - | KC992562 | KC935140 |
| Enochrus fimbriatus | KC935258 | KC992456 | KC992668 | KC935034 | KC992563 | KC935141 |
| Enochrus mastersii | KC935259 | KC992472 | - | KC935035 | - | - |
| Enochrus ochraceus | KC935260 | KC992481 | KC992669 | KC935036 | KC992564 | KC935142 |
| Enochrus piceus | KC935261 | KC992428 | KC992670 | KC935037 | KC992565 | - |
| Enochrus quadripunctatus | AM287090 | AM287112 | AM287068 | AM287127 | AM287135 | - |
| Enochrus sp. | KC935262 | KC992485 | KC992671 | KC935038 | KC992566 | KC935143 |
| Enochrus talamanca | KC935263 | KC992486 | KC992672 | KC935039 | KC992567 | KC935144 |
| Enochrus testaceus | AM287089 | AM287111 | AM287067 | AJ810719 | AJ810754 | - |
| Epimetopus sp. | AM287082 | AM287104 | AM287060 | AJ810724 | AJ810759 | - |
| Epimetopus sp. | KC935264 | KC992452 | KC992673 | KC935040 | KC992568 | KC935145 |
| Gen. nov. | KC935282 | KC992488 | - | KC935062 | KC992588 | KC935163 |
| Georissus canalifer | KC935265 | KC992401 | KC992674 | KC935041 | KC992569 | KC935146 |
| Georissus crenulatus | DQ221983 | - | DQ202580 | AY745584 | - | - |
| Georissus sp. | - | AM287105 | - | - | AJ810751 | - |
| Globulosis sp. | KC935266 | KC992433 | KC992675 | KC935042 | KC992570 | KC935147 |
| Guyanobius lacuniventris | KC935267 | KC992406 | KC992676 | KC935043 | KC992571 | KC935148 |
| Guyanobius sp. | KC935268 | KC992405 | KC992677 | KC935044 | KC992572 | - |
| Helobata sp. | KC935269 | KC992453 | KC992678 | KC935045 | KC992573 | KC935149 |
| Helochares sp. | KC935270 | KC992471 | - | KC935046 | KC992574 | KC935150 |
| Helochares sp. | KC935271 | KC992460 | KC992679 | KC935047 | KC992575 | - |
| Helocombus bifidus | KC935272 | KC992475 | - | KC935048 | KC992576 | KC935151 |
| Helophorus aquaticus | AM287078 | AM287100 | AM287056 | AJ810741 | AJ810749 | - |
| Helophorus arvernicus | AM287079 | AM287101 | AM287057 | AM287122 | AM287130 | - |
| Helophorus guttulus | AM278080 | AM287102 | AM287058 | AM287123 | AM287131 | - |
| Helophorus nivalis | AM287081 | AM287103 | - | - | AJ810750 | - |
| Hemiosus selva | - | KC992412 | KC992680 | KC935049 | KC992577 | KC935152 |
| Hemisphaera seriatopunctata | KC935273 | KC992427 | - | KC935050 | KC992578 | KC935153 |
| Horelophopsis hanseni | KC935274 | - | - | KC935051 | KC992579 | KC935154 |
| Horelophus walkeri | KC935275 | KC992430 | - | KC935052 | KC992580 | KC935155 |
| Horelophus walkeri | KC935276 | KC992431 | KC992681 | KC935053 | KC992581 | KC935156 |
| Hybogralius hartmeyeri | KC935277 | - | - | KC935054 | KC992582 | KC935157 |
| Hydrobiomorpha spinicollis gr. | KC935278 | KC992418 | KC992682 | KC935055 | KC992583 | KC935158 |
| Hydrobius fuscipes | KC935279 | KC992497 | - | KC935056 | KC992584 | KC935159 |
| Hydrobius fuscipes | AM287092 | AM287114 | AM287070 | AJ810720 | AJ810755 | - |
| Hydrobius melaenus | KC935280 | KC992495 | - | KC935057 | KC992585 | - |
| Hydrochara obtusata | KC935281 | - | - | KC935058 | KC992586 | KC935160 |
| Hydrochus angustatus | HM569417 | - | - | AF427601 | - | - |
| Hydrochus carinatus | AM287084 | AM287106 | AM287062 | AM287124 | AM287132 | - |
| Hydrochus elongatus | HM569423 | - | - | AJ810717 | AJ810752 | - |
| Hydrochus spangleri | - | KC992415 | KC992683 | KC935059 | KC992587 | KC935161 |
| Hydrophilomima sp. | - | KC992438 | KC992686 | KC935063 | KC992589 | KC935164 |
| Hydrophilus albipes | - | - | - | KC935064 | KC992590 | KC935165 |
| Hydrostygnus frontalis | KC935285 | KC992434 | KC992687 | KC935066 | KC992591 | KC935167 |
| Kanala montaguei | KC935286 | KC992512 | KC992688 | - | - | KC935168 |
| Laccobius reflexipennis | KC935287 | KC992439 | KC992689 | KC935067 | KC992592 | KC935169 |
| Laccobius serratus | KC935288 | KC992450 | KC992690 | KC935068 | KC992593 | KC935170 |
| Limnoxenus niger | KC935289 | KC992493 | - | KC935069 | KC992594 | KC935171 |
| Limnoxenus semicylindricus | KC935290 | KC992496 | KC992691 | KC935070 | KC992595 | KC935172 |
| Limnoxenus zealandicus | KC935291 | KC992494 | - | KC935071 | KC992596 | KC935173 |
| Moraphilus sp. | KC935292 | KC992457 | - | KC935072 | KC992597 | - |
| Notionotus liparus | KC935293 | - | - | KC935073 | KC992598 | KC935174 |
| Notionotus sp. | KC935294 | KC992508 | KC992692 | KC935074 | KC992599 | KC935175 |
| nr. Tobochares sp. | KC935283 | KC992487 | KC992685 | KC935060 | KC992601 | KC935162 |
| nr. Tobochares sp. | - | KC992474 | KC992684 | KC935061 | KC992600 | - |
| Omicrogiton insularis | KC935295 | - | - | KC935075 | KC992602 | - |
| Oocyclus funestus | KC935297 | KC992445 | KC992693 | KC935076 | KC992603 | KC935176 |
| Oocyclus madidus | KC935298 | KC992435 | - | KC935077 | KC992604 | KC935177 |
| Oocyclus ornatus | KC935299 | KC992446 | KC992694 | - | KC992605 | KC935178 |
| Oocyclus petra | KC935300 | KC992442 | KC992695 | KC935078 | KC992606 | KC935179 |
| Oocyclus pico | KC935301 | KC992447 | KC992696 | - | KC992607 | KC935180 |
| Oocyclus sapphirus | KC935302 | KC992444 | KC992697 | - | KC992608 | KC935181 |
| Oocyclus sp. | KC935303 | KC992436 | KC992698 | - | KC992609 | KC935182 |
| Oocyclus sp. | KC935304 | KC992437 | - | - | KC992610 | KC935183 |
| Oocyclus trujillo | KC935305 | KC992448 | KC992699 | - | KC992611 | KC935184 |
| Oosternum soricoides gr. | KC935306 | KC992513 | - | KC935079 | KC992612 | - |
| Opthalmocyclus sp. | KC935307 | KC992443 | KC992700 | KC935080 | KC992613 | KC935185 |
| Pachysternum nigrovittatum | KC935308 | KC992492 | - | KC935081 | KC992614 | - |
| Pacrillum sp. | KC935309 | KC992489 | - | KC935082 | KC992615 | - |
| Paracymus limbatus | KC935310 | KC992425 | KC992701 | KC935083 | KC992616 | KC935186 |
| Paracymus pygmaeus | KC935311 | KC992473 | KC992702 | KC935084 | KC992617 | KC935187 |
| Paracymus subcupreus | KC935312 | - | - | KC935085 | KC992618 | KC935188 |
| Paroosternum saundersi | - | - | - | KC935086 | KC992619 | KC935189 |
| Pelthydrus minutus gr. | KC935313 | KC992422 | - | KC935087 | KC992620 | - |
| Peratogonus sp. | KC935314 | KC992408 | - | KC935088 | KC992621 | - |
| Peratogonus sp. | - | KC992407 | - | KC935089 | KC992622 | KC935190 |
| Phaenonotum caribense | KC935315 | KC992399 | - | KC935090 | KC992623 | - |
| Protosternum sp. | - | KC992403 | KC992703 | KC935091 | KC992624 | KC935191 |
| Psalitrus sp. | KC935316 | KC992410 | - | KC935092 | KC992625 | - |
| Regimbartia attenuata | KC935317 | KC992423 | KC992704 | KC935093 | KC992626 | KC935192 |
| Rygmodus sp. | KC935318 | KC992505 | KC992705 | KC935094 | KC992627 | KC935193 |
| Saphydrus sp. | KC935319 | KC992509 | KC992706 | KC935095 | KC992628 | KC935194 |
| Scoliopsis sp. | KC935320 | KC992476 | KC992707 | KC935096 | KC992629 | KC935195 |
| Scoliopsis sp. | KC935321 | KC992477 | KC992708 | KC935097 | KC992630 | KC935196 |
| Spercheus emarginatus | AM287085 | AM287107 | KC992709 | AJ810718 | AJ810753 | KC935197 |
| Sperchopsis tessellata | KC935322 | KC992421 | - | KC935098 | KC992631 | KC935198 |
| Sphaeridium bipustulatum | AM287095 | KC992459 | KC992710 | AJ810722 | AJ810757 | KC935199 |
| Sphaeridium bipustulatum | KC935323 | KC992458 | - | KC935099 | - | KC935200 |
| Sphaeridium lunatum | KC935324 | - | - | KC935100 | - | KC935201 |
| Sternolophus marginicollis | KC935325 | KC992416 | KC992711 | KC935101 | KC992632 | KC935202 |
| Thysanarthria sp. | KC935326 | KC992432 | KC992712 | KC935102 | KC992633 | KC935203 |
| Tobochares sulcatus | KC935327 | KC992465 | KC992713 | KC935103 | KC992634 | KC935204 |
| Tormus sp. | KC935328 | - | - | KC935104 | KC992635 | - |
| Tropisternus affinis | KC935329 | - | - | KC935105 | KC992636 | KC935205 |
| Tropisternus collaris | KC935330 | KC992419 | KC992714 | KC935106 | KC992637 | KC935206 |
| Tropisternus lateralis | KC935331 | KC992417 | KC992715 | KC935107 | KC992638 | KC935207 |
| Tylomicrus costatus | KC935332 | KC992426 | - | - | KC992639 | KC935208 |
